# Circ_0001825 promotes osteogenic differentiation in human-derived mesenchymal stem cells via miR-1270/SMAD5 axis

**DOI:** 10.1186/s13018-023-04133-5

**Published:** 2023-09-06

**Authors:** Changjun Zheng, Lingzhi Ding, Ziming Xiang, Mingxuan Feng, Fujiang Zhao, Zhaoxin Zhou, Chang She

**Affiliations:** 1 Department of Joint Surgery, The Second Affiliated Hospital of Soochow University, No. 1055, Sanxiang Road, Suzhou, 215004 Jiangsu Province China; 2 Department of Orthopedics, Taizhou Central Hospital (Taizhou University Hospital), Taizhou, 318000 Zhejiang China

**Keywords:** circ_0001825, miR-1270, SMAD5, Osteoporosis, Osteogenic differentiation

## Abstract

**Background:**

The implication of deregulated circular RNAs in osteoporosis (OP) has gradually been proposed. Herein, we aimed to study the function and mechanism of circ_0001825 in OP using osteogenic-induced human-derived mesenchymal stem cells (hMSCs).

**Methods:**

The content of genes and proteins was tested by quantitative real-time polymerase chain reaction and Western blotting. The osteogenic differentiation in hMSCs were evaluated by ALP activity and Alizarin Red staining, as well as the detection of osteogenesis-related markers. Cell viability and apoptosis were measured by CCK-8 assay and flow cytometry. The binding between miR-1270 and circ_0001825 or SMAD5 (SMAD Family Member 5) was confirmed by using dual-luciferase reporter assay and pull-down assay.

**Results:**

Circ_0001825 was lowly expressed in OP patients and osteogenic induced hMSCs. Knockdown of circ_0001825 suppressed hMSC viability and osteogenic differentiation, while circ_0001825 overexpression showed the exact opposite effects. Mechanistically, circ_0001825/miR-1270/SMAD5 formed a feedback loop. MiR-1270 was increased and SMAD5 was decreased in OP patients and osteogenic induced hMSCs. MiR-1270 up-regulation suppressed hMSC viability and osteogenic differentiation, which was reversed by SMAD5 overexpression. Moreover, miR-1270 deficiency abolished the effects of circ_0001825 knockdown on hMSCs.

**Conclusion:**

Circ_0001825 promoted hMSC viability and osteogenic differentiation via miR-1270/SMAD5 axis, suggesting the potential involvement of circ_0001825 in osteoporosis.

**Supplementary Information:**

The online version contains supplementary material available at 10.1186/s13018-023-04133-5.

## Introduction

Osteoporosis (OP) is a systemic skeletal disorder featured by defective bone architecture and low bone mineral density (BMD), leading to the susceptibility to fractures [1]. OP has high morbidity especially in postmenopausal women, and accounts for about 80% of all fractures in clinic that principally threatens the life quality and increases disability, premature mortality and financial burden [2, 3]. Despite advances in therapeutics, OP remains an incurable chronic condition [4]. Nevertheless, pharmacological interventions are available, and it is still urgently to deeply understand the pathogenesis of OP to develop effective methods.

As a kind of noncoding molecules, circular RNAs (circRNAs) possesses a covalent closed-loop structure that lacks 5′–3′ ends, so they are more stability compared with other linear RNAs [5]. Besides that, the regulatory functions of circRNAs in diverse biological processes, such as cell proliferation, apoptosis, differentiation and glycolysis, have been identified [6–8]. For example, circRNA_0000285 was detected to accelerate cervical cancer cell migration and growth abilities via down-regulating FUS [9]. CircCTNNB1 alleviated cerebral ischemia/reperfusion (I/R) injury by negatively affecting apoptotic, inflammatory and oxidative injury via miR-96-5p/SRB1 axis [10]. Meanwhile, growing proofs have indicated that most circRNAs are differentially expressed in several bone diseases, and broadly participate in osteogenic differentiation and OP pathogenesis [11, 12]. CircRNAs are considered as ideal biomarkers for developing molecular targeting treatment for OP [12]. Circ_0001825 is new-found circRNA originated from ZFAT gene in chr8: 135521861–135545215, through the microarray dataset GSE161361, the expression of circ_0001825 was detect to be decreased in OP samples. However, the functions of circ_0001825 in the pathogenesis of OP have not yet been reported.

Mesenchymal stem cells (MSCs) are one kind of undifferentiated cells with multi-linage differentiation capabilities and can differentiate into adipocytes, osteoblasts, or chondrocytes, the senescence of mesenchymal stem cells (MSCs) has a significant role during the pathogenesis of OP [13]. Herein, this study used human-derived MSCs (hMSCs) to investigate the role of circ_0001825 in osteogenic differentiation. Moreover, some circRNAs can function as microRNA (miRNA) sponges to modulate the biological functions and expression profile of the downstream genes [14, 15]. Thus, the potential miRNA/mRNA axis of circ_0001825 in hMSCs was also explored.

## Material and methods

### Clinical samples

Bone marrow samples were collected from trauma patients (27 postmenopausal OP patients and 23 age- and gender-matched postmenopausal women without OP (Control)) in the Second Affiliated Hospital of Soochow University and Taizhou Central Hospital. The OP patients were diagnosed based on the World Health Organizations diagnostic criteria [16]. All samples were preserved at − 80 °C for analysis. The study was approved by the Ethics Committee of the Second Affiliated Hospital of Soochow University and Taizhou Central Hospital. It was performed in accordance with the Declaration of Helsinki. It has obtained the informed consents from all individuals.

### Cell culture and differentiation

Human-derived mesenchymal stem cells (hMSCs) was obtained from American Tissue Culture Collection (ATCC, Manassas, VA, USA) and cultured in growth medium (ATCC) containing hMSC basal medium, 1% penicillin–streptomycin and fetal bovine serum (FBS) at 37 °C with 5% CO_2_. For the induction of osteogenesis, hMSCs were maintained in differentiation medium consisting of DMEM low glucose medium, 10 mM β-glycerol phosphate, 50 ng/ml L-ascorbic acid, 0.1 µM dexamethasone (Sigma-Aldrich, St. Louis, MO, USA) and 10% FBS for up to 14 days. The medium is renewed every 3 days.

### Quantitative real-time PCR (qRT-PCR)

The TRIzol reagent (Invitrogen, Carlsbad, CA, USA) was adopted to extract total RNAs. The synthesis of cDNA was conducted by reverse transcription using Prime Script RT Reagent Kit (Takara, Dalian, China), and the amplification reaction was performed using SYBR Green kit (Takara) through qRT-PCR analysis. GAPDH or U6 was used as the control and the relative folds changes were calculated by 2^–ΔΔCt^ method. Primers were shown in Table 1.

**Table 1:** Primers sequences used in this study

Name	Primers for qRT-PCR (5′–3′)
*hsa_circ_0001825*	
Forward	TGGTTAAGCAGCCTTTCCGC
Reverse	CTGTTGTAGTGCCGCTTCAG
*SMAD5*	
Forward	TCGGATGTACCACCCTGGAT
Reverse	TTCCTTTCGATAAGCGCGGA
*miR-1270*	
Forward	GTATGAGCTGGAGATATGGAAGAG
Reverse	CTCAACTGGTGTCGTGGAG
*miR-1287-5p*	
Forward	GTATGAGTGCTGGATCAGTGGTT
Reverse	CTCAACTGGTGTCGTGGAG
*GAPDH*	
Forward	GGAGCGAGATCCCTCCAAAAT
Reverse	GGCTGTTGTCATACTTCTCATGG
*U6*	
Forward	GCTTCGGCAGCACATATACTAA
Reverse	AACGCTTCACGAATTTGCGT

### RNase R and actinomycin D assay

For the stability analysis of circ_0001825, about 3 µg isolated RNAs were used to incubate with RNase R (5 U/μg) or Mock for 20 min at 37 °C, and the resulting RNA was collected qRT-PCR analysis.

In addition, hMSCs were treated with 2 μg/ml Actinomycin D for indicated times, then RNAs were isolated from cells and the contents of circular and linear RNAs were tested using qRT-PCR.

### Cell transfection

Two specific short hairpin RNAs (shRNAs) targeting circ_0001825 (sh-circ_0001825#1 and sh-circ_0001825#2), and miR-1270 mimics or inhibitor (miR-1270 or anti-miR-1270) were constructed by Genema (Shanghai, China) with nontraget shRNA (sh-NC), miR-NC or anti-miR-NC as the contrasts. The full-length of circ_0001825 or SMAD5 was cloned into pCD5-ciR or pcDNA3.1 plasmids (Genema) to establish overexpression vectors with empty plasmids as the control (pCD5-ciR or pcDNA). Then Lipofectamine 2000 (Invitrogen) was applied to conduct transient transfection.

### Cell counting kit-8 (CCK-8) assay

hMSCs were planted in 96-well plates, and incubated for 24, 48 or 72 h. Then 10 μL CCK-8 solution (Beyotime, Beijing, China) was supplemented into each well for an additional 2 h-incubation. Finally, the absorbance at 450 nm was assessed by a microplate reader.

### Flow cytometry

hMSCs were harvested and washed by PBS, then cells were suspended in binding buffer (400 μL) containing 10 µL Annexin V-FITC and 5 µL PI. After incubation for 15 min in darkness, apoptosis cells were examined by a flow cytometry.

### Alkaline phosphatase (ALP) staining and activity detection

hMSCs were immobilized with 1% Triton X-100 (Sigma) for 15 min. After being centrifuged at 14,000×*g* for 5 min, cell supernatant were collected and then dyed with ALP colorimetric assay kit (BioVision, Milpitas, CA, USA). Finally, the absorbance at 405 nm was assessed by a microplate reader to calculate the activity of ALP. In addition, histochemical ALP staining were conducted as per the protocol of a commercial ALP staining Kit (GeFan Biotech, shanghai, China) with the supernatant.

### Alizarin red staining

hMSCs were harvested and washed by 1X PBS for twice, followed by being fixed in 10% formalin for 20 min. Next, cells were dyed with 1 mL 0.5% alizarin red staining solution for 15 min at indoor temperature. Following washing with distilled water for 5 min, cells were transferred to slides, and red mineralized nodules were observed and analyzed using a microscope at 572 nm.

### Western blotting

Total proteins were isolated and separated by 10% SDS-PAGE gels. The proteins were then shifted onto PVDF membranes (Merck Millipore, Billerica, MA, USA). Then the primary antibodies against osteopontin (OPN; ab8448), osteocalcin (OCN; ab93876), SMAD5 (ab40771) and GAPDH (ab8245) were used to incubate with the membranes all night at 4 °C, followed by secondary incubation with HRP-conjugated secondary antibodies for 2 h at 37 °C. All antibodies were obtained from Abcam (Cambridge, MA, USA). Proteins were observed using the ECL procedure (Millipore).

### Dual-luciferase reporter assay

The fragments of miR-1270 on circ_0001825 and SMAD5 CDS region were amplified and inserted into the pMIR-reporter (Promega, Beijing, China) to establish wild-type (WT) vectors (WT-circ_0001825 or WT-SMAD5 CDS). Then the mutation (MUT) sites of seed sequence was designed, and MUT-circ_0001825 or MUT-SMAD5 CDS vector was established. Then the 200 ng recombinant vectors and 50 nM miR-1270 or miR-NC were transfected into hMSCs, and the luciferase activity levels were determined after 48 h.

### Pull-down assay

Biotinylated miR-1270 (bio-miR-1270) or the control (bio-miR-NC) was designed and established, and then incubated with 2 μg cell lysate of hMSCs. Then the mixture was reacted with 100 μL Streptavidin C1 magnetic beads (Invitrogen) for 1 h. Finally, the enrichments of circ_0001825 and SMAD5 were tested by qRT-PCR.

### Statistical analyses

The results were manifested as mean ± standard deviation (SD). Group comparison was conducted by analysis of variance in multiple groups, or Student’s t test in two groups. GraphPad Prism 9 (GraphPad Software, San Diego, CA, United States) were used for statistical analysis. *P* < 0.05 indicated significant differences.

## Results

### Circ_0001825 was lowly expressed in OP patients and osteogenic induced hMSCs

**Fig. 1:**
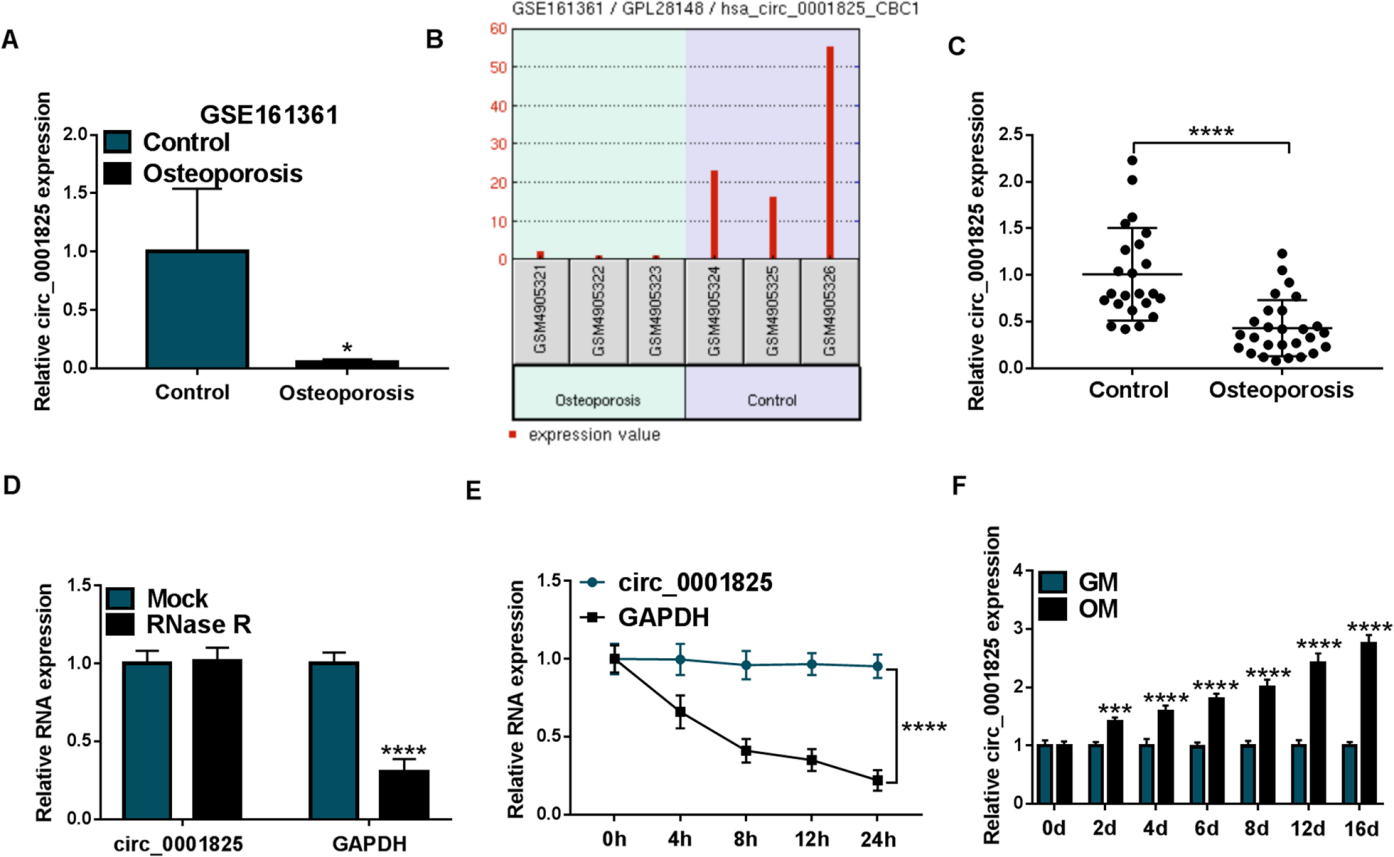
Circ_0001825 was lowly expressed in OP patients and osteogenic induced hMSCs. **a, b** Circ_0001825 was lowly expressed in OP patients as compared to normal control in GSE161361 database. **c** qRT-PCR analysis of circ_0001825 expression in bone marrow samples of OP patients and normal contrasts. **d, e** Cyclization validation of circ_0001825 by RNase R and actinomycin D treatment. **f** qRT-PCR analysis of circ_0001825 expression in hMSCs incubated with osteogenic medium (OM) or growth medium (GM). *P < 0.05; ***P < 0.001; ****P < 0.0001

Through the microarray dataset GSE161361 (https://www.ncbi.nlm.nih.gov/geo/geo2r/?acc=GSE161361), the expression of circ_0001825 was identified to be down-regulated in OP samples (3 pairs of OP and matched normal tissues) (Fig. 1a, b). Then the expression profile of circ_0001825 in clinical samples of OP was evaluated. As exhibited in Fig. 1c, circ_0001825 was lowly expressed in the bone marrow samples of OP patients relative to the normal control. Next, it was discovered that RNase R treatment could digest linear GAPDH but not affect circ_0001825 in hMSCs (Fig. 1d). Further actinomycin D treatment also showed that circ_0001825 was stable relative to linear GAPDH mRNA (Fig. 1e), indicating the cyclization characteristics of circ_0001825. Besides that, qRT-PCR analysis showed that circ_0001825 level was increased in hMSCs incubated under osteogenic medium (OM) than that in cell incubated with growth medium (GM) (Fig. 1f). These data suggested that down-regulated circ_0001825 might be associated with OP progression by affecting osteogenic differentiation.

### Circ_0001825 knockdown suppressed viability and osteogenic differentiation in hMSCs

**Fig. 2:**
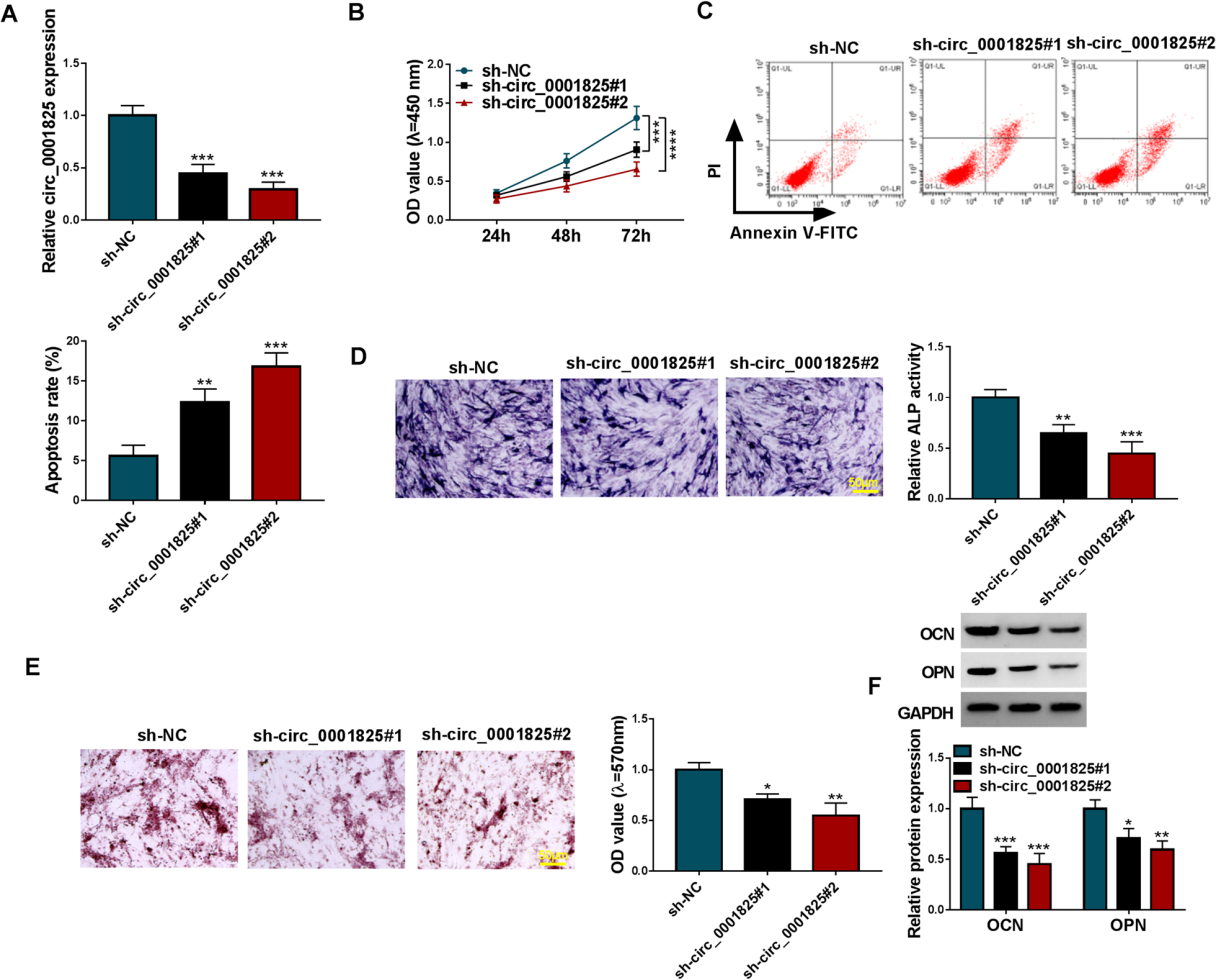
Circ_0001825 knockdown suppressed viability and osteogenic differentiation in hMSCs. **a–f** hMSCs at 14 days of osteogenic induction were transfected with sh-NC or sh-circ_0001825 (sh-circ_0001825#1 and sh-circ_0001825#2). **a** qRT-PCR analysis for circ_0001825 content. **b** CCK-8 assay for cell viability. **c** Flow cytometry for cell apoptosis. **d–f** Osteogenic differentiation was evaluated by ALP activity, or Alizarin Red staining and the detection of osteogenesis-related markers (OCN and OPN). **P < 0.01; ***P < 0.001

To validate the function of circ_0001825 in OP. Loss-of-function assay [17, 18] was conducted by using circ_0001825 specific shRNAs (sh-circ_0001825#1 and sh-circ_0001825#2). hMSCs at 14 days of osteogenic induction were exposed to assigned transfection. qRT-PCR analysis showed both sh-circ_0001825#1 and sh-circ_0001825#2 transfection markedly decreased circ_0001825 expression in hMSCs compared with sh-NC introduction (Fig. 2a). Functionally, deficiency of circ_0001825 suppressed cell viability (Fig. 2b) and induced apoptosis (Fig. 2c) in hMSCs. In addition, circ_0001825 deletion weakened the activity of ALP in hMSCs (Fig. 2d and Additional file 1: Fig. S1A). Furthermore, alizarin red staining exhibited that calcium deposition was increased in sh-circ_0001825 group compared with corresponding controls (Fig. 2e and Additional file 1: Fig. S1B). Besides that, western blotting showed the decreases of osteogenesis-related markers (OCN and OPN) in hMSCs at protein levels (Fig. 2f). In all, circ_0001825 knockdown suppressed viability and osteogenic differentiation in hMSCs.

### Circ_0001825 overexpression promoted viability and osteogenic differentiation in hMSCs

**Fig. 3:**
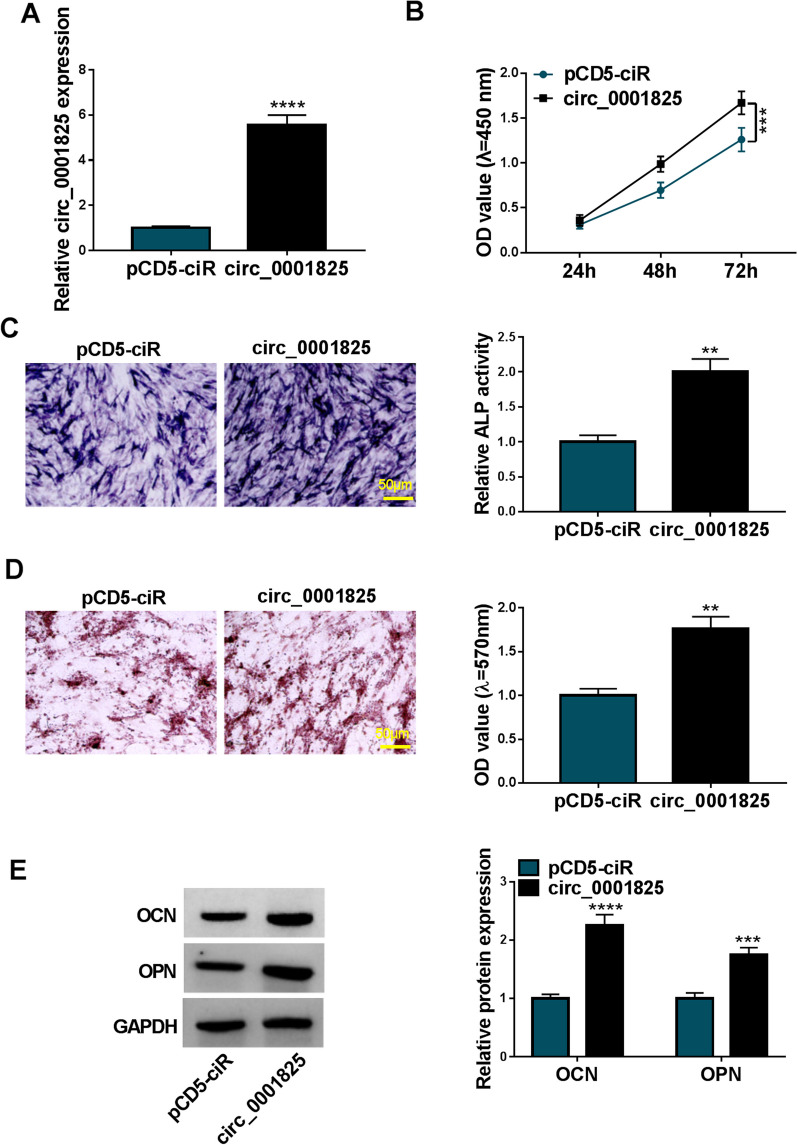
Circ_0001825 overexpression promoted viability and osteogenic differentiation in hMSCs. **a–e** hMSCs at 14 days of osteogenic induction were transfected with circ_0001825 or pCD5-ciR. **a** qRT-PCR analysis for circ_0001825 content. **b** CCK-8 assay for cell viability. **c–e** Osteogenic differentiation was evaluated by ALP activity, or Alizarin Red staining and the detection of osteogenesis-related markers (OCN and OPN). **P < 0.01; ***P < 0.001

Next, gain-of-function assay was performed. hMSCs subjected to osteogenic induction for 14 d, and then transfected with circ_0001825 overexpression plasmids and empty plasmids. As expected, circ_0001825 level was markedly elevated after circ_0001825 plasmids introduction (Fig. 3a). Thereafter, we discovered that circ_0001825 overexpression led to the promotion of cell viability (Fig. 3b). Moreover, ALP activity and calcium deposition were increased after circ_0001825 overexpression (Fig. 3c, d). Also, circ_0001825 overexpression enhanced the protein levels of OCN and OPN in hMSCs (Fig. 3e). Thus, circ_0001825 contributed to hMSC viability and osteogenic differentiation.

### MiR-1270 was a target of circ_0001825

**Fig. 4:**
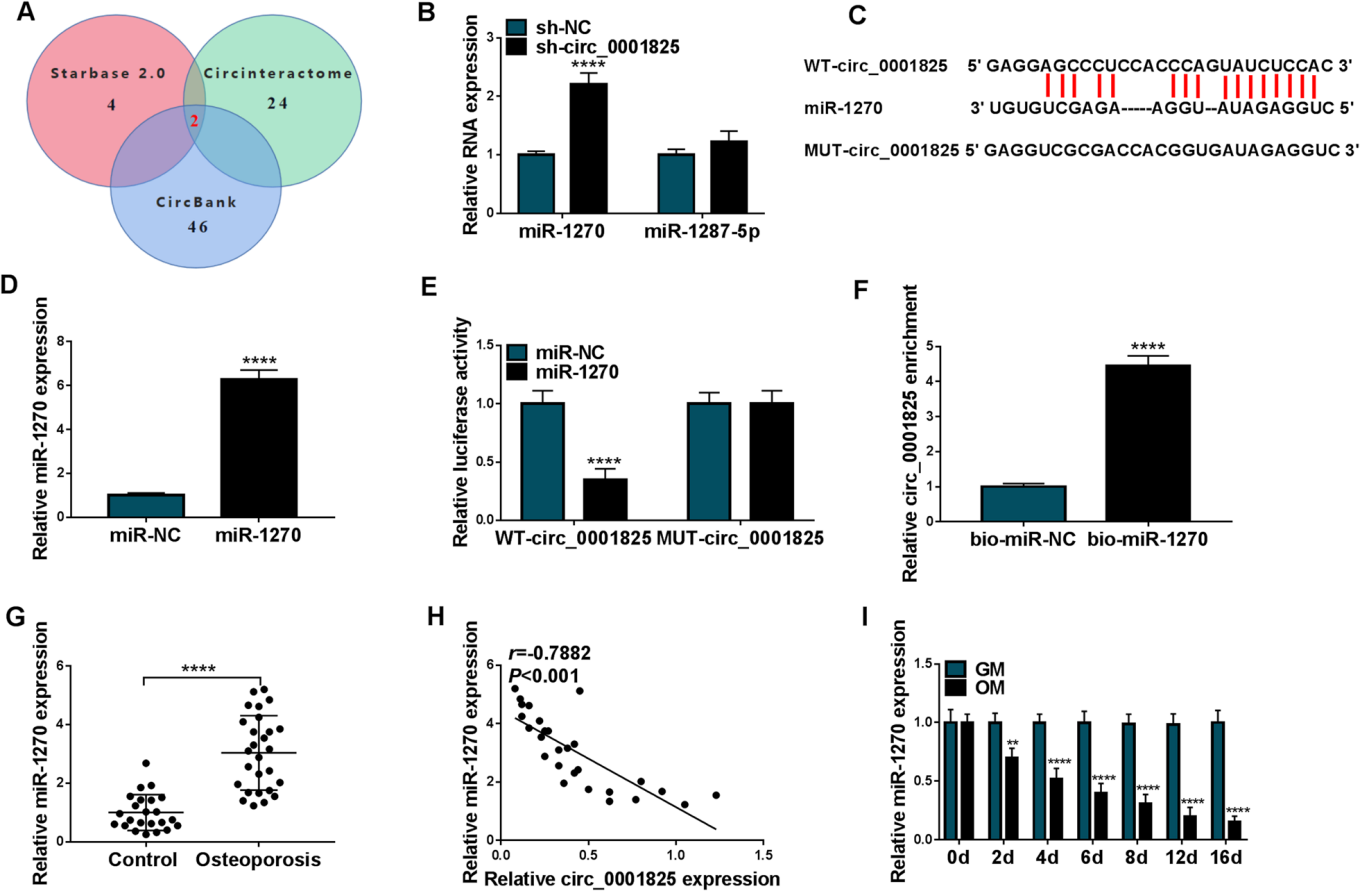
MiR-1270 was a target of circ_0001825. **a** Schematic illustration exhibiting overlapping of the target miRNAs of circ_0001825 predicted by Starbase2.0, circBank and Circinteractome databases. **b** qRT-PCR analysis for the expression of miR-1270 and miR-1287-5p in hMSCs transfected with sh-NC or sh-circ_0001825. **c** The binding sites of miR-1270 on circ_0001825. **d** Transfection efficiency detection by qRT-PCR. **e**, **f** The interaction analysis between miR-1270 and circ_0001825 by dual-luciferase reporter assay and pull-down assay. **g** qRT-PCR analysis of miR-1270 expression in bone marrow samples of OP patients and normal contrasts. **h** Correlation analysis between miR-1270 and circ_0001825 expression in OP samples. **i** qRT-PCR analysis of miR-1270 expression in hMSCs incubated with OM or GM. **P < 0.01; ****P < 0.0001

Subsequently, the underlying targets of circ_0001825 were investigated. Three databases (Starbase2.0, circBank and Circinteractome) were used to predict the potential target miRNAs, among which 2 miRNAs (miR-1270 and miR-1287-5p) were selected from the overlap between the databases (Fig. 4a). Meanwhile, it was found that circ_0001825 knockdown led to significant elevation of miR-1270 levels in hMSCs, but did not affect miR-1287-5p expression in cells (Fig. 4b). Thus, miR-1270 might be a target of circ_0001825. Figure 4c showed the binding sites of miR-1270 on circ_0001825. After confirming the overexpression efficiency of miR-1270 mimic (Fig. 4d), dual-luciferase reporter assay was carried out. The results showed that miR-1270 up-regulation markedly weakened the luciferase activity in WT-circ_0001825 group, but not the MUT-circ_0001825 group in hMSCs (Fig. 4e). Furthermore, pull-down assay suggested that circ_0001825 was notably captured by biotinylated miR-1270 compared with the control in hMSCs (Fig. 4f). In addition, miR-1270 was highly expressed in the bone marrow samples of OP patients relative to the normal control (Fig. 4g), and was negatively correlated with circ_0001825 expression (Fig. 4h). Moreover, miR-1270 level was decreased in hMSCs incubated with OM than that in cell incubated with GM (Fig. 4i). Collectively, circ_0001825 directly targeted miR-1270.

### Circ_0001825 regulated the viability and osteogenic differentiation in hMSCs via miR-1270

**Fig. 5:**
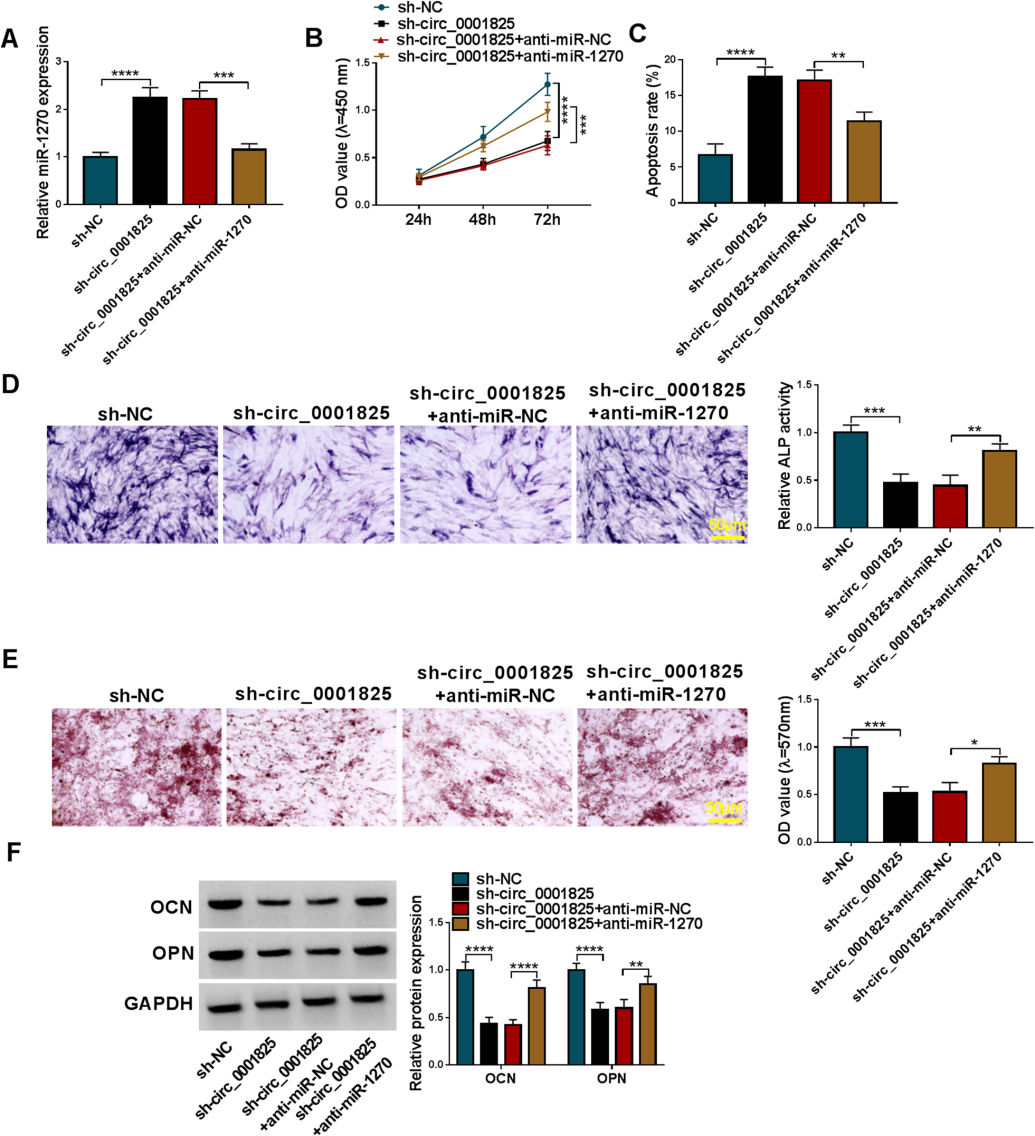
Circ_0001825 regulated the viability and osteogenic differentiation in hMSCs via miR-1270. **a–f** hMSCs were transfected with sh-circ_0001825 alone or sh-circ_0001825 together with anti-miR-1270 after osteogenic induction. **a** qRT-PCR analysis for circ_0001825 content. **b** CCK-8 assay for cell viability. **c** Flow cytometry for cell apoptosis. **d–f** Osteogenic differentiation was evaluated by ALP activity, or Alizarin Red staining and the detection of osteogenesis-related markers (OCN and OPN). *P < 0.05; **P < 0.01; ***P < 0.001; ****P < 0.0001

To further study whether miR-1270 was a functional target of circ_0001825, hMSCs were transfected with sh-circ_0001825 (sh-circ_0001825#1) alone or sh-circ_0001825 together with miR-1270 inhibitor (anti-miR-1270) after osteogenic induction. qRT-PCR analysis showed anti-miR-1270 introduction reversed sh-circ_0001825-induced increase of miR-1270 level in hMSCs (Fig. 5a). Then we found the inhibition of cell viability and enhancement of cell apoptosis mediated by sh-circ_0001825 were rescued by anti-miR-1270 introduction (Fig. 5b, c). Moreover, miR-1270 inhibition abolished sh-circ_0001825-caused inhibition on osteogenic differentiation, evidenced by reduced ALP activity, calcium deposition, and protein levels of OCN and OPN in hMSCs (Fig. 5d–f). Altogether, circ_0001825 promoted hMSC viability and osteogenic differentiation via miR-1270.

### SMAD5 was a target of miR-1270

**Fig. 6:**
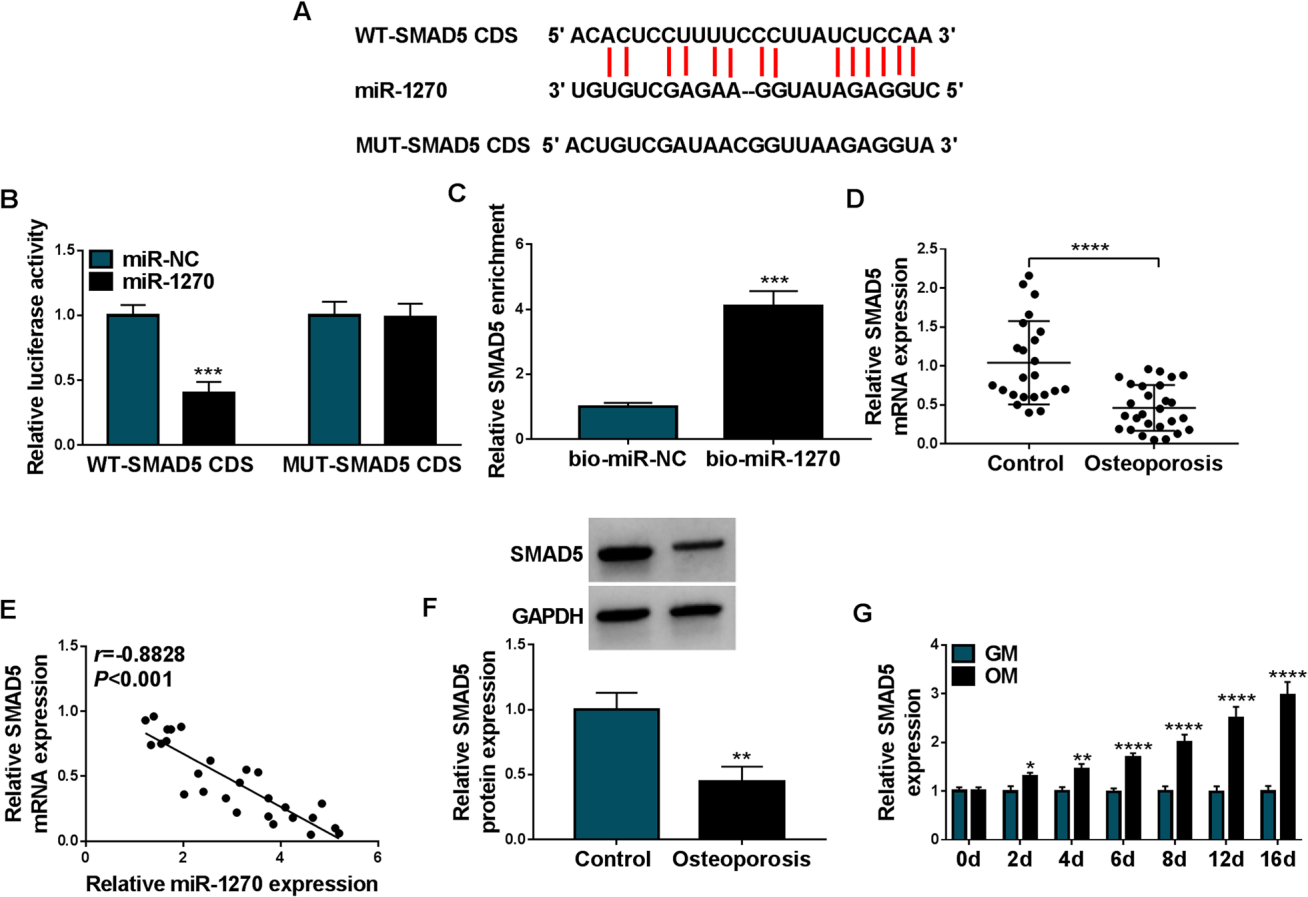
SMAD5 was a target of miR-1270. **a** The binding sites of miR-1270 on SMAD5. **b, c** The interaction analysis between miR-1270 and SMAD5 by dual-luciferase reporter assay and pull-down assay. **d** qRT-PCR analysis of SMAD5 expression in bone marrow samples of OP patients and normal contrasts. **e** Correlation analysis between miR-1270 and SMAD5 expression in OP samples. **f** Western blotting analysis of SMAD5 expression in bone marrow samples of OP patients and normal contrasts. **g** qRT-PCR analysis of SMAD5 expression in hMSCs incubated with OM or GM. *P < 0.05; **P < 0.01; ***P < 0.001; ****P < 0.0001

Then we further explored the targets of miR-1270. Through the prediction of starBase v2.0 database, miR-1270 was found to have binding sites on SMAD5 (Fig. 6a). Dual-luciferase reporter assay showed that miR-1270 mimic markedly weakened the luciferase activity of WT-SMAD5 CDS, and did not affect the luciferase activity of MUT-SMAD5 CDS in hMSCs (Fig. 6b). Also, pull-down assay also confirmed the binding between miR-1270 and SMAD5 (Fig. 6c). The mRNA level of SMAD5 was decreased in OP samples (Fig. 6d), which was negatively correlated with miR-1270 expression (Fig. 6e). Similarly, the protein levels of SMAD5 also showed decreasing trend in OP patients (Fig. 6f). Importantly, OM maintain led to an increase of SMAD5 expression level compared with GM culture (Fig. 6g). These data verified miR-1270 targeted SMAD5.

### MiR-1270 suppressed the viability and osteogenic differentiation in hMSCs via SMAD5

**Fig. 7:**
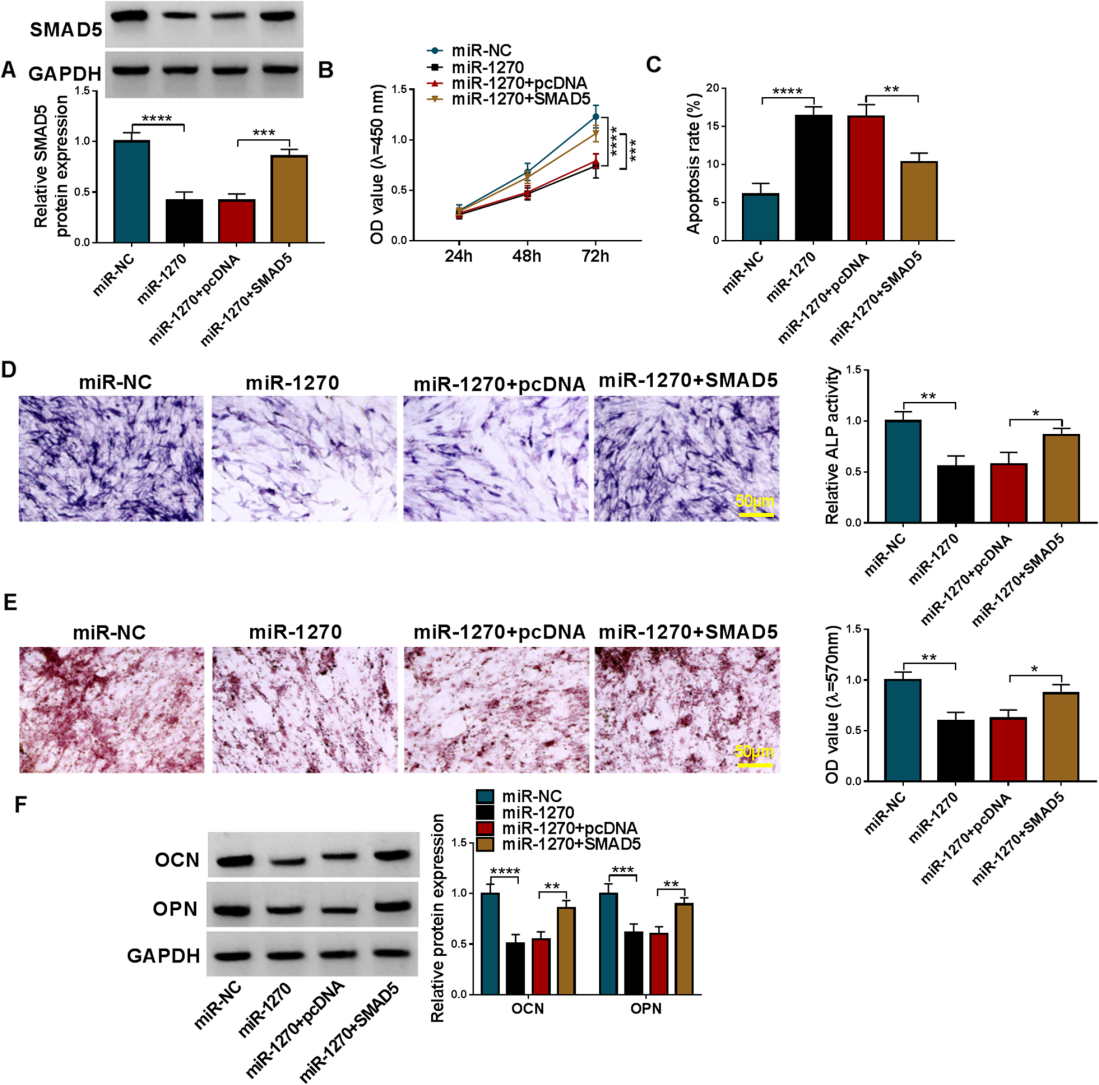
MiR-1270 suppressed the viability and osteogenic differentiation in hMSCs via SMAD5. **a–f** hMSCs were transfected with miR-1270 alone or miR-1270 together with SMAD5 after osteogenic induction. **a** Western blotting analysis for SMAD5 content. **b** CCK-8 assay for cell viability. **c** Flow cytometry for cell apoptosis. **d–f** Osteogenic differentiation was evaluated by ALP activity, or Alizarin Red staining and the detection of osteogenesis-related markers (OCN and OPN). *P < 0.05; **P < 0.01; ***P < 0.001; ****P < 0.0001

Thereafter, the potential functions of miR-1270/SMAD5 axis on OP were explored. hMSCs were transfected with miR-1270 alone or miR-1270 together with SMAD5 after osteogenic induction. Western blotting analysis showed that miR-1270 mimic resulted in a decrease of SMAD5 level, which was rescued by SMAD5 plasmids transfection (Fig. 7a). Functionally, miR-1270 mimic suppressed the viability and induced apoptosis in hMSCs, while these effects were abated by SMAD5 up-regulation (Fig. 7b, c). Besides that, miR-1270 mimic weakened osteogenic differentiation in hMSCs, reflected by decreased ALP activity, calcium deposition, and protein levels of OCN and OPN in hMSCs, while SMAD5 plasmids transfection counteracted the effects mediated by miR-1270 mimic (Fig. 7d–f). In short, miR-1270 suppressed the viability and osteogenic differentiation in hMSCs via SMAD5.

### Circ_0001825/miR-1270/SMAD5 formed a feedback loop

**Fig. 8:**
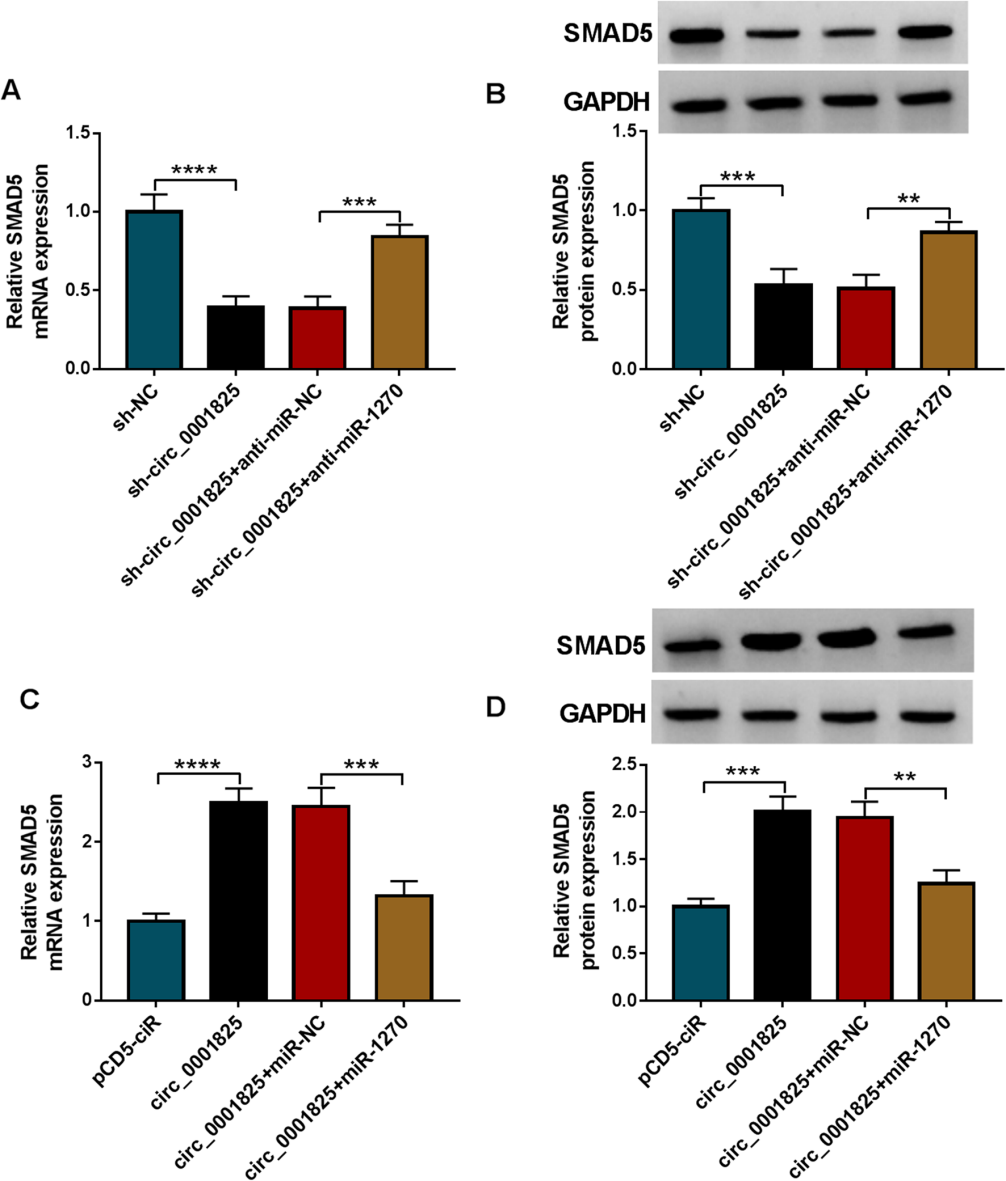
Circ_0001825/miR-1270/SMAD5 formed a feedback loop. **a–d** qRT-PCR and western blotting were used to evaluate the effects of circ_0001825/miR-1270 axis on the expression change of SMAD5. **P < 0.01; ***P < 0.001; ****P < 0.0001

As shown in Fig. 8a, b, circ_0001825 silencing was accompanied with decreased SMAD5 level, which was rescued by following miR-1270 inhibition in hMSCs. Moreover, the level of SMAD5 was elevated by circ_0001825 overexpression, and then reduced in response to following miR-1270 mimic addition (Fig. 8c, d). Therefore, there was a circ_0001825/miR-1270/SMAD5 axis in hMSCs.

## Discussion

Currently, drug-based agents that induce osteogenesis or suppress bone resorption remain the mainstream therapy for OP, whereas, they are not able to reverse the loss of existing bones and may also result in side effects such as cancer, osteonecrosis of the jaw, or thromboembolic events [19, 20]. Therefore, exploring alternative therapeutic strategies for OP are urgent. Currently, the study on molecularly targeted therapy have attracted great research interest. CircRNAs have been considered as ideal biomarkers for developing molecule-based therapeutics due to their high stability and tissue- or cell type-specific expression [21]. Moreover, a large number of circRNAs have been reported to modulate osteoblast differentiation, thereby affecting OP progression. For example, circRNA_0048211 and circ_0001795 induced osteoblastic differentiation in hMSCs to attenuate OP progression [22, 23]. However, hsa_circ_0006859 was confirmed to stimulate adipogenesis and inhibit osteogenic differentiation in hMSCs [24]. In addition, circRNA_28313 deficiency suppressed osteoclast differentiation in bone marrow monocyte/macrophage cells and impaired bone resorption in vivo [25]. Herein, we found circ_0001825 was lowly expressed in OP patients and osteogenic-induced hMSCs. Functionally, circ_0001825 overexpression promoted hMSC visibility and weakened cell apoptosis. Moreover, circ_0001825 up-regulation in hMSCs elevated the expression of osteogenesis-associated genes and induced mineralization ability and ALP activity. While circ_0001825 deficiency showed opposite effects. Therefore, circ_0001825 may be a potential biomarker for regulating postmenopausal OP.

In a further mechanical analysis, the miRNA/mRNA axis underlying circ_0001825 in hMSCs was identified based on the ceRNA hypothesis [14, 15], and the circ_0001825/miR-1270/SMAD5 axis was first identified. MiRNAs are broadly expressed and powerful regulators of complex processes, accumulating evidence has identified the potential of miRNAs in the therapeutics of many diseases [26, 27]. A previous study exhibited that miR-1270 was overexpressed in circulating monocytes of postmenopausal OP patients and was associated with bone remodeling genes [28]. Besides that, Eric Gustavo’s team showed that miR-1270 attenuated the proliferation and mobility of osteoblastic cell lines, implying the association between miR-1270 and bone metabolism [29]. In our work, OP patients and osteogenic-induced hMSCs also showed high expression of miR-1270. Overexpression of miR-1270 in hMSCs suppressed the viability and osteogenesis in cells. Moreover, miR-1270 silencing abated the suppressing effects of circ_0001825 deficiency on osteogenic differentiation in hMSCs. SMAD5 is a member of the receptor-activated Smad that act as critical signal transducers of TGF-β-related signaling [30]. SMAD5-mediated signals govern osteogenesis through the comprehensive interactions with RUNX2, SIP1 and Rob [31]. Yan et al*.* showed that miR-222-3p reduced the levels of SMAD5 and RUNX2, thereby weakening osteogenic differentiation in hMSCs [32]. Wei’s team suggested that SMAD5 inhibition was accompanied with RUNX2 decrease, and then suppressed osteogenic differentiation, which might be related to the miR-21-SMAD5 axis [33]. In our data, we found a decrease of SMAD5 content in OP patients and osteogenic-induced hMSCs. Rescue assay indicated that enforced expression of SMAD5 reversed the suppressing functions of miR-1270 on hMSC viability and osteogenesis.

In all, our data first demonstrated that circ_0001825 contributed to the viability and osteogenic differentiation in hMSCs via activating SMAD5 through sequestering miR-1270, providing a new insight into the development of circRNA-based therapeutics in osteoporosis intervention. In addition, MSC are mesoderm-derived undifferentiated cells, which can alos differentiate into chondrogenic or adipogenic cells under the in vivo or in vitro stimulation. Thus, we will explore interactions of adipogenesis and chondrogenesis with circ_0001825 in the future, and further elaborate the role of circ_0001825 in bone metabolic diseases.

### Supplementary Information

**Additional file 1: Fig. S1**. Related to Fig. 2

**Additional file 2: Fig. S2**. Related to Fig. 3

**Additional file 3: Fig. S3**. Related to Fig. 5

**Additional file 4: Fig. S4**. Related to Fig. 7. The gross appearance of ALP and Alizarin Red staining by naked eyes. (A) ALP staining. (B) Alizarin Red staining.

## Data Availability

The data sets used and/or analyzed during the current study are available from the corresponding author on reasonable request.
