# Supplementary figures and images for: Circ_0001825 promotes osteogenic differentiation in human-derived mesenchymal stem cells via miR-1270/SMAD5 axis
Source: J Orthop Surg Res. 2023 Sep 6;18:663. doi: 10.1186/s13018-023-04133-5 (PMC10481475; doi:10.1186/s13018-023-04133-5)

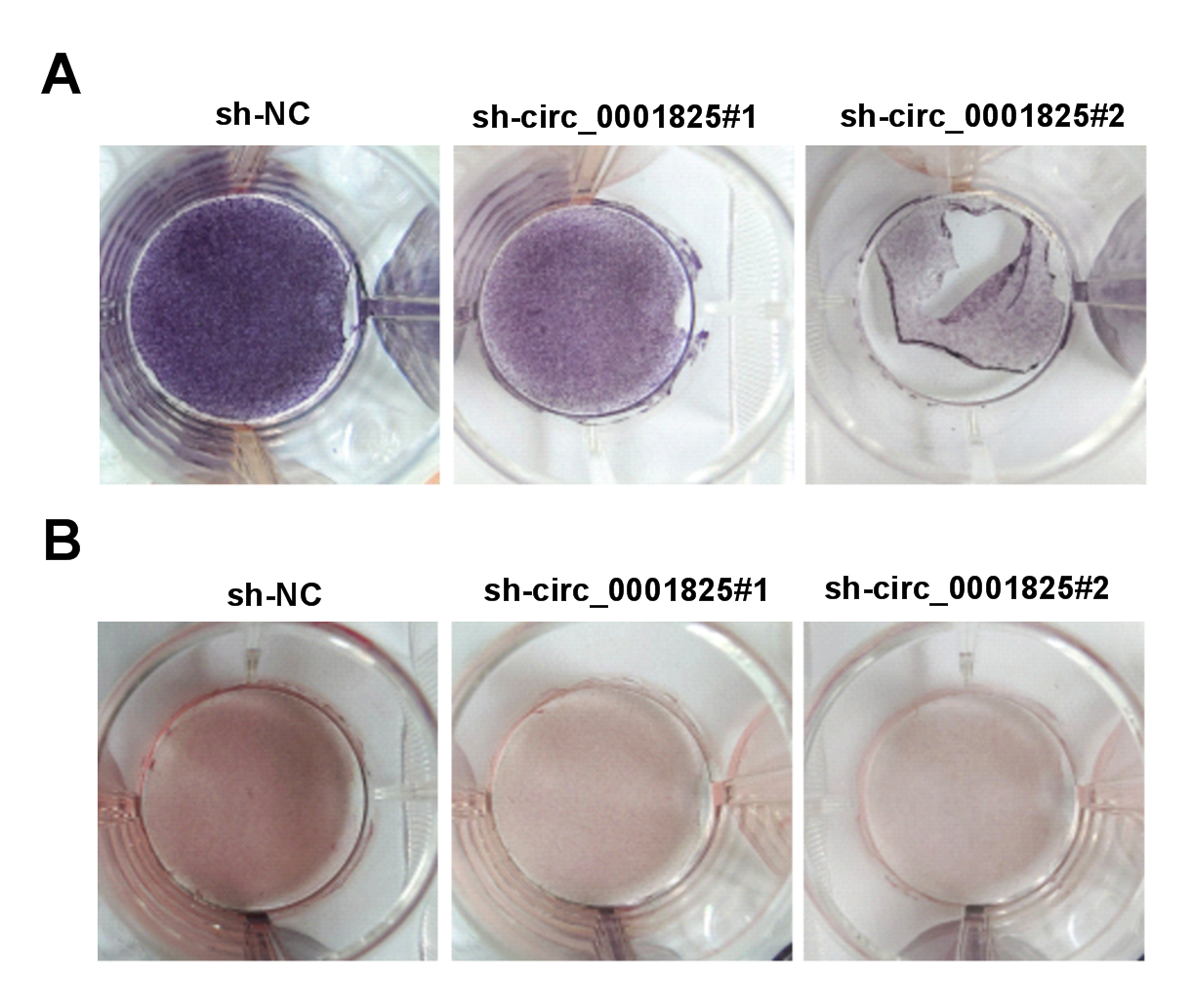

Supplement: Supplementary file 1 — Additional file 1: Fig. S1. Related to Fig. 2 [file 13018_2023_4133_MOESM1_ESM.tif]

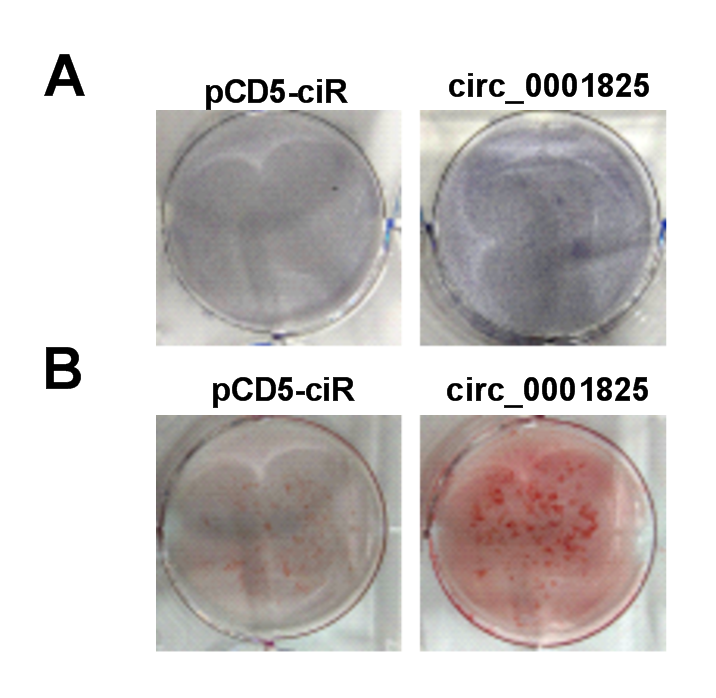

Supplement: Supplementary file 2 — Additional file 2: Fig. S2. Related to Fig. 3 [file 13018_2023_4133_MOESM2_ESM.tif]

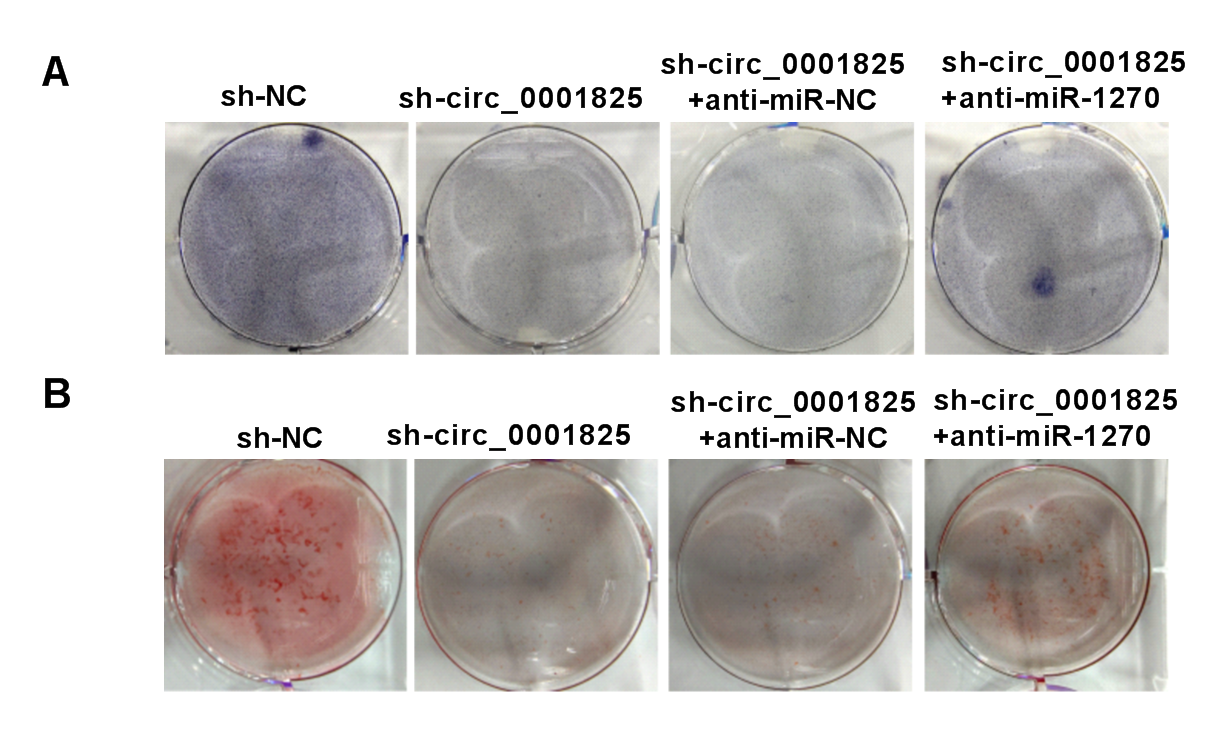

Supplement: Supplementary file 3 — Additional file 3: Fig. S3. Related to Fig. 5 [file 13018_2023_4133_MOESM3_ESM.tif]

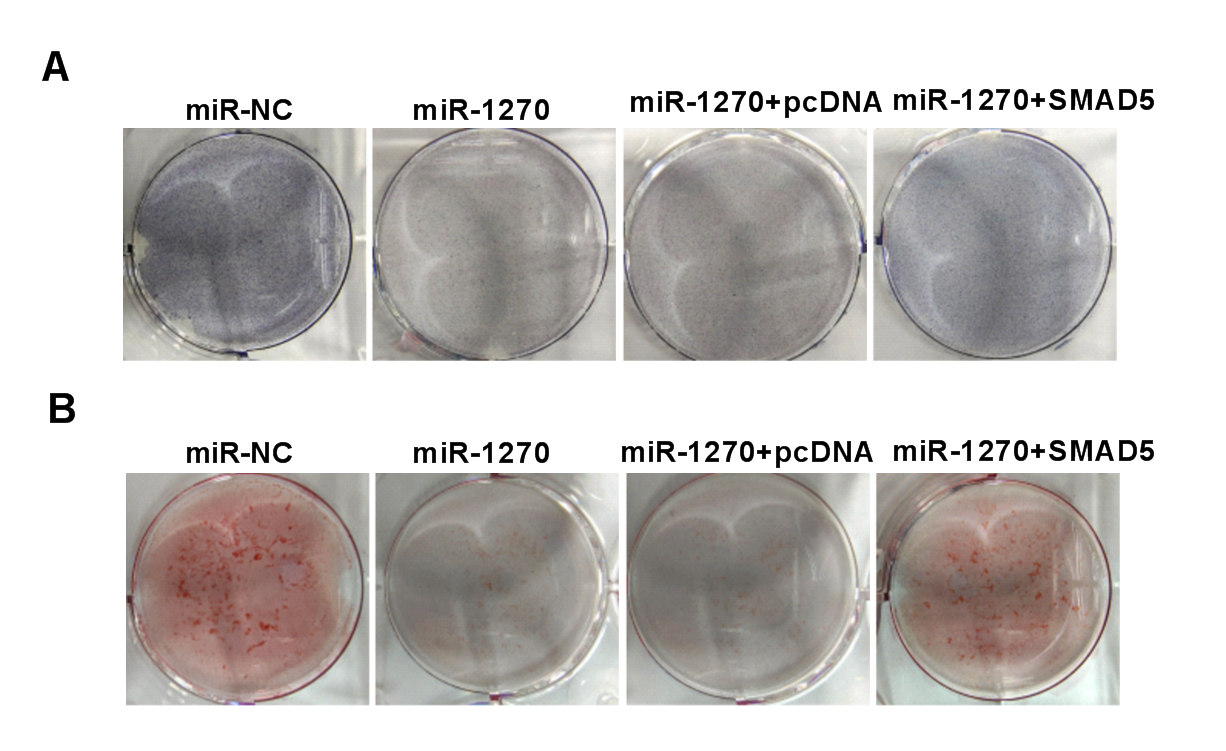

Supplement: Supplementary file 4 — Additional file 4: Fig. S4. Related to Fig. 7. The gross appearance of ALP and Alizarin Red staining by naked eyes. (A) ALP staining. (B) Alizarin Red staining. [file 13018_2023_4133_MOESM4_ESM.tif]
